# Is it time to shorten the blanking period after atrial fibrillation ablation procedures?

**DOI:** 10.1016/j.ahjo.2025.100523

**Published:** 2025-03-05

**Authors:** Gerald V. Naccarelli

**Affiliations:** Penn State University College of Medicine, Penn State Hershey Heart & Vascular Institute, 500 University Drive, Room H1511, Hershey, PA 17033, USA

**Keywords:** Atrial fibrillation, Blanking periods, Catheter ablation of atrial fibrillation

Early recurrence of atrial tachyarrhythmia (ERAT) following pulmonary vein isolation (PVI) catheter ablation of atrial fibrillation (AF), can occur in up to 65 % in the first three months after the procedure depending on the electrocardiographic monitoring strategy [1,2]. Older age, male gender, structural heart disease, longer AF duration, higher CHA₂DS₂-VASc scores, larger left atrial size and left ventricular LV dysfunction predict ERAT and late AF recurrence [1,2].

ERAT is attributed to transient inflammation from the tissue damage during ablation and short-term imbalances in autonomic innervation [[1], [2], [3]]. AF triggers not originating from the pulmonary veins are another source of ERAT and long-term AF recurrences. Consensus statements have supported a three-month blanking period after AF ablation although the most recent consensus statement suggests shortening the blanking period to eight weeks [3].

Pulmonary vein ablation line reconnections can occur within days of the procedure and express themselves after the local edema from the ablation has resolved [[1], [2], [3]]. In addition, ERAT can be secondary to a new proarrhythmic response from the ablation such as left atrial reentrant tachycardia. ERAT is not considered a failure of the ablation procedure since the mechanism is different and early recurrences are not always predictive of long-term AF recurrences [2,4]. Patients without ERAT have excellent long-term efficacy from their ablation procedure [4]. ERAT appears to be independent of the type of energy used for the PVI.

A clinical issue using blanking periods is that ERAT may not ameliorate over time. The occurrence of ERAT during the blanking period is associated with a lower PVI one-year success rate of 38.1 % compared to 79.5 % in those without ERAT (*p* < .001) [5]. ERAT burdens of twenty-three minutes or longer have a seven-fold increase in AF recurrences after the blanking period [6]. ERAT that occurs later in the blanking period is a predictor of long-term AF relapses. Implantable loop recorder studies have reported that recurrences of atrial tachyarrhythmias after 52 days have a 95 % predictive rate of later AF recurrences [7]. Management of symptomatic ERAT is an issue since repeat ablations are usually reserved for patients with recurrences after the blanking period or for symptomatic persistent atrial flutter or left atrial tachycardias that occur early and cannot be treated pharmacologically. Symptomatic ERAT is usually treated with a combination of rate and rhythm control medications. It is common to continue antiarrhythmic drugs after a PVI to minimize early atrial arrhythmia recurrences. If effective, this minimizes arrhythmic recurrences but does this approach mask the true efficacy of the ablation procedure. Early recurrences can be ignored or treated with antiarrhythmic agents and direct current cardioversion with presumed efficacy yet a single recurrence on an antiarrhythmic drug is considered a failed attempt at rhythm control. This has been an issue in comparing a catheter ablation strategy to an antiarrhythmic drug strategy in prospective trials such as CABANA [8].

In this issue of the American Heart Journal Plus, Ruzieh et al. [9] discussed potential problems of using blanking periods including the interpretation of the results of comparative trials. A key point of their commentary is that blanking periods might not affect hard endpoints like mortality but the effect on arrhythmia recurrences biases efficacy endpoints towards the ablation procedure. Longer blanking periods artificially add to the presumed efficacy of the PVI procedure. We should not count early recurrences of AF during drug therapy when the drug is being titrated or has not reached steady state. Except for amiodarone, steady state for other antiarrhythmic drugs occurs within four days. In the case of amiodarone, recurrences of AF within the first months are expected since the drug has not reached steady state. However, adverse effects that are thought to occur from amiodarone such as bradycardia, AV block, gastrointestinal issues are counted against the drug and changes are made based on these.

Are blanking periods fair for assessing safety? We do not use a blanking period for adverse effects related to a PVI procedure or other common forms of ablation. Obviously, mechanical adverse effects such as cardiac perforation, cardiac tamponade or embolic stroke immediately caused by the ablation procedure do not need a blanking period but what about complications that may occur weeks after a PVI like deep venous thrombosis, pulmonary embolus or esophageal fistula?

Are there any solutions to the blanking period dilemma? Does the blanking period have to be 3 months in duration? Over twenty years ago, Oral et al. reported that most early recurrences occur in the first two weeks and inflammatory markers are only increased for a week after ablation procedures [10]. Basic models of ablation have shown that local edema resolves within 4 weeks [3]. Instead of eliminating the blanking period, an alternative would be to shorten the period to four to eight weeks after the procedure. Arguments for this approach include meta-analysis data reporting that ERAT after the first month (27.7 days) was associated with a 76 % predictive AF recurrence rate [11]. In the ADVICE trial, one year freedom of AF recurrences after the blanking period was time sensitive to the timing of ERAT with recurrences of 62.6 % at one-month, 36.4 % at two-months and 7.8 % at 3 months [12]. This study supports the recent consensus statement suggesting an 8-week blanking period [2].

Blanking periods for AF ablation tip the scales to increase efficacy and minimize adverse events compared to other rhythm control therapies. In CABANA, although 24-h Holter monitors were obtained monthly in the first six months, only the 96-h monitor data at six months was reported [8]. Ruzieh et al. [9] suggest reporting all arrhythmia recurrences in trial no matter when they occur, and I support this suggestion.

I agree with the recent consensus statement that shortening the blanking period to eight would minimize the issue and be more consistent with basic physiology and the bulk of the timing of these arrhythmic recurrences. Shortening this period would have little effect on the day-to-day management of patents with ERAT and minimize the length of blanking periods used in future AF ablation trials.

## CRediT authorship contribution statement

**Gerald V. Naccarelli:** Conceptualization, Writing – original draft, Writing – review & editing.

## Declaration of competing interest

Gerald V. Naccarelli MD (Consultant: Acesion)
